# Study on the loss of load-carrying capacity of thin-walled composite columns with closed cross-sections

**DOI:** 10.1038/s41598-025-26003-2

**Published:** 2025-11-26

**Authors:** Patryk Rozylo

**Affiliations:** Department of Machine Design and Mechatronics, Faculty of Mechanical Engineering, Lublin University of Technology, Nadbystrzycka 36, Lublin, 20-618 Poland

**Keywords:** Composite structures, Experimental studies, Numerical simulations, Progressive failure analysis, Failure of composites, Engineering, Materials science, Mathematics and computing

## Abstract

The subject of the study was thin-walled composite columns with a closed cross-section. The aim of the research was to analyse the failure of compressed thin-walled sections with three cross-section shapes and four composite lay-ups. The study investigated the effects of column cross-section shape and composite lay-up on loss of load-carrying capacity. Experimental studies of the axial compression of thin-walled composite structures were conducted using interdisciplinary research methods, thereby determining, among other things, post-buckling equilibrium paths and modes of structural failure. In numerical simulations, the research was conducted using original finite element method models, including a failure model based on progressive failure analysis. The novelty of the present paper was the evaluation of the load capacity of profiles with closed sections, with particular attention to the influence of cross-section shape and composite-ply arrangement. The experimental and numerical results of the study were in quantitative and qualitative agreement.

## Introduction

Thin-walled composite structures are a special group of load-carrying structures that are used in the automotive, construction, or, above all, aerospace industries. Structures of this type are characterized by the fact that they undergo the phenomenon of buckling, followed by a change in the form of deformation under axial compression^1,2^. With further loading of composite structures (after buckling), the structure enters the post-buckling range of its load-deflection curve, ultimately leading to failure^3–6^. Post-buckling equilibrium paths represent experimental curves that describe the load-displacement relationship throughout the entire test (from the initial loading phase to failure). It is important to thoroughly evaluate the failure loads on composite structures^7–10^. The above is possible through interdisciplinary research techniques^11^. In experimental research, the phenomenon of failure is analysed using a universal testing machine^11^ and additional apparatus, such as an optical deformation measurement system (DIC)^6,8^, acoustic emission^7^, or a digital microscope with a mobile working head. The use of the above-mentioned testing methods enabled the determination of post-buckling equilibrium paths over the full load range, recording structural failure modes and detecting acoustic emission signals, thereby improving the identification of the damage mechanism^12–15^. As part of parallel numerical simulations, research was carried out using the finite element method. The numerical simulations included solving the nonlinear structural stability problem using progressive failure analysis^1^. The use of progressive failure analysis enables the assessment of composite structure failure, evaluating whether failure occurs due to fibre or matrix tension or compression, or layered shear. The present study focused solely on the phenomenon of failure, which manifested as complex damage to the composite material (ply cracking, delamination)^5^. In failure studies, it is important to understand the nature of progressive damage to the structure, and independent experimental and numerical testing methods are helpful for a thorough assessment of this condition. The novelty of the present study lay in the object of research itself - thin-walled profiles with closed sections made of CFRP material, produced for the realization of the research project funded by the National Science Centre, Reg. No. 2021/41/B/ST8/00148, and the complex analysis of the influence of lay-up and cross-section shape on the failure of the structure. The investigations presented in this publication constitute a summary of the research conducted in the above-mentioned scientific project, aimed at assessing the load-carrying capacity of composite structures with a closed cross-section.

## The subject of study

**Fig. 1:**
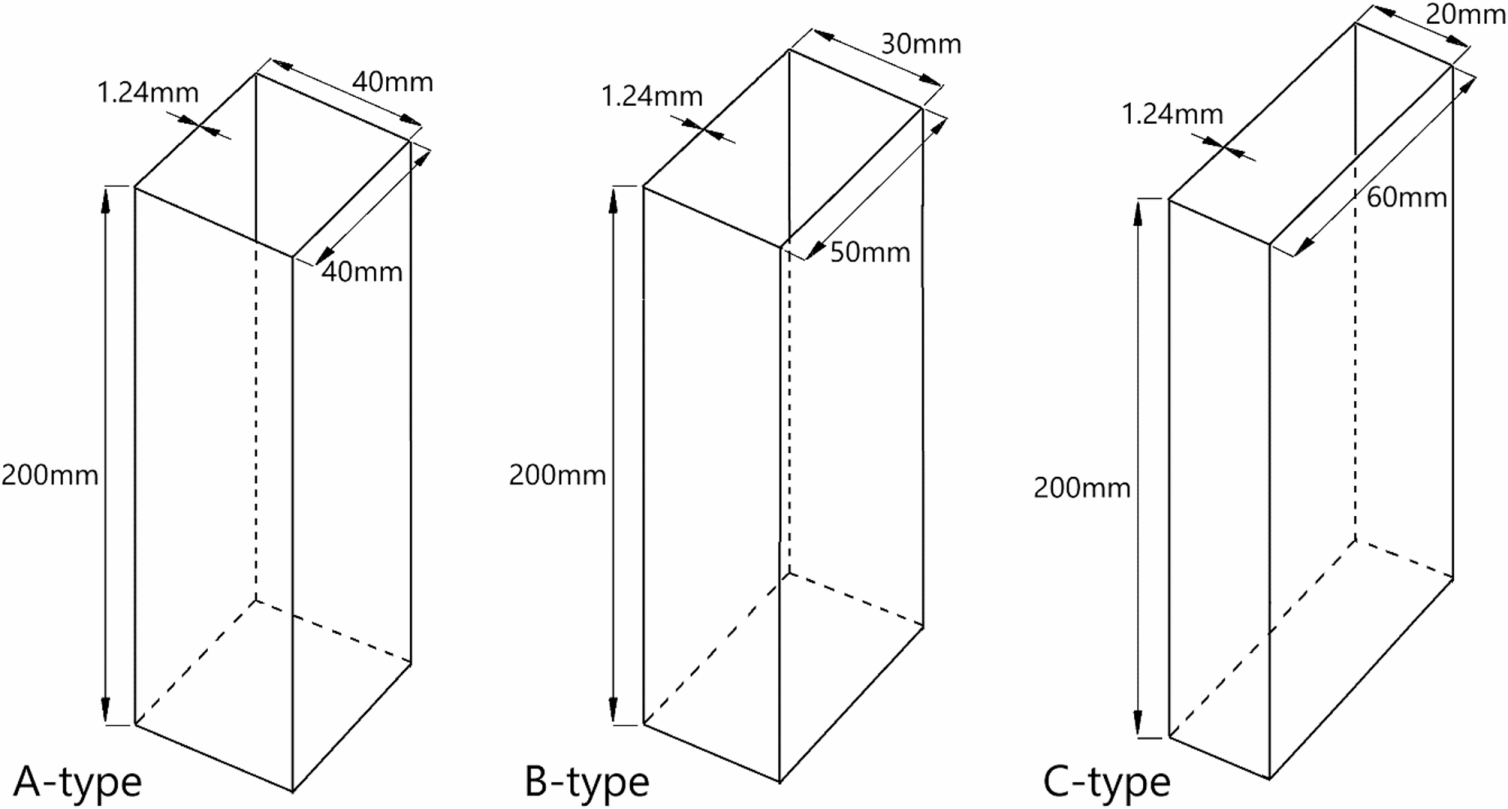
Test specimens with different cross-sections.

The study focused on thin-walled composite profiles manufactured by a private company (WIT-Composites) for a scientific project. Profiles produced by the autoclave technique were prepared from CYCOM 985 − 42%-HS-135-305 TENAX HTA prepreg, in which the CYCOM resin system accounted for 42% of the material volume. The high-strength fibres used in the TENAX HTA prepreg had a fibre weight (grammage) of 135 g/m^2^. Tenax^®^ HTA Carbon Fibre is a high-performance carbon fibre produced by Teijin Limited. It exhibits high tensile strength and modulus, making it well-suited for applications requiring low weight and high stiffness. The composite material included unidirectional (UD) reinforcement, a type of composite reinforcement in which all carbon fibres are aligned in the same direction (within a single ply). All profiles were cured in an autoclave at 177 °C and at an over-pressure of 0.6 MPa. Composite profiles were manufactured as described in publications^16,17^. The profiles consisted of 8 plies with a total thickness of 1.24 mm. The geometric parameters of the profiles are presented in detail in Fig. 1. Three cases of cross-sectional area (internal cross-section) of the composite profiles were considered in the study, i.e., A − 40 × 40 mm, B − 50 × 30 mm, and C − 60 × 20 mm. Four different arrangements of plies (lay-ups) characterized the test profiles, i.e., 1 - [0°/45°/-45°/90°]_s_, 2 - [0°/90°/0°/90°]_s_, 3 - [45°/-45°/90°/0°]_s_, 4 - [90°/-45°/45°/0°]_s_. There were 3 test specimens for each of the laminate lay-up configurations, making a total of: 4 lay-ups x 3 cross-section types x 3 test specimens = 36 specimens. For example, the A1 profile designation constitutes a structure with cross-section type A 40 × 40 mm and the first lay-up system 1 - [0°/45°/-45°/90°]_s_. The test specimens, regardless of cross-section shape (types A, B, and C), had identical circumference due to the equal sum of the lengths of the edges forming the cross-section.

All properties of the composite material were determined in accordance with ISO standards: PN-EN ISO 527–5 – static tensile test, PN-EN ISO 14,129 – static shear test, PN-EN ISO 14,126 – static compression test. All necessary material properties (both mechanical and strength) were determined based on static strength tests. The procedure for determining the composite material’s material properties was presented in detail in a previous research paper^18^. The aforementioned publication presents a methodology for determining the properties of the composite material presented in this paper. A detailed explanation of the coefficients of CFRP (carbon fiber reinforced polymer) presented in Table 1 was provided in^18^.

**Table 1:** Composite material properties (CFRP) – average values (with standard deviation)^18^.

Mechanical Properties		Strength Properties	
Young’s modulus *E*_1_ [GPa]	103.01 (± 2.15)	Tensile Strength *F*_TU_ *(0°)* [GPa]	1.28 (± 0.06)
Young’s modulus *E*_2_ [GPa]	7.36 (± 0.31)	Compressive Strength *F*_CU_ *(0°)* [GPa]	0.57 (± 0.05)
Poisson’s ratio *v*_12_ [-]	0.37 (± 0.17)	Tensile Strength *F*_TU_ *(90°)* [GPa]	0.03 (± 0.01)
Kirchhoff modulus *G*_12_ [GPa]	4.04 (± 0.17)	Compressive Strength *F*_CU_ *(90°)* [GPa]	0.10 (± 0.01)
-	-	Shear Strength *F*_SU_ *(45°)* [GPa]	0.13 (± 0.01)

The determined material properties made it possible to further include them in numerical models as part of parallel simulations using FEM. This approach will enable relatively similar research results between experimental tests and numerical simulations. The selection of the geometric parameters of the columns was determined by their practical application as load-carrying elements, e.g. in the construction industry in public-use structures. It was important to maintain certain common features, such as wall thickness, composite column height and identical sum of edge lengths forming cross-sections. This made it possible to estimate which type of structure would be less susceptible to damage. In addition, the layer arrangements used were based on previous research on composite structures with open cross-sections, as presented in other scientific publications^1,11^. The selected layer arrangements were characterised by identical fibre angles, i.e. 0°, 45° and 90°, with different combinations of layer arrangements in a symmetrical arrangement, within 8 layers per composite structure. The selected layer arrangements were estimated at the beginning of the research project with the National Science Centre in the context of preliminary FEM simulations.

### Experimental studies

Experimental research was carried out using four independent testing devices. The basic machine for carrying out experimental tests of axial compression was a Zwick Z100 universal testing machine^11^. During the experimental tests, the axial compression conditions of the composite columns were provided. The influence of boundary condition imperfections was reduced to a minimum. For this purpose, special supports with flat working surfaces were used, between which the test specimens were mounted. Throughout the duration of the tests, the parallelism between the support elements was maintained. Load and displacement values were recorded during the tests, enabling the generation of post-buckling equilibrium paths for thin-walled composite structures. The recorded value of the displacement of the machine head (to which the cross-section was adjacent during the entire duration of the tests) was a value that corresponded approximately to the shortening of the composite specimens. Experimental tests were conducted at a constant traverse speed of 1 mm/min at room temperature.

Another device used in the experimental study was an optical system for measuring structural deformation - the Aramis 2D version^19,20^. This system uses a technique commonly known as digital image correlation (DIC). The measurement is non-contact and independent of the materials under test. The user can leverage both whole-surface data analysis and point-method data. The digital image correlation system Aramis enables experimental testing on real objects with relatively different geometric parameters, depending on the system version. In the course of the conducted stability tests involving axial compression of the structure, local forms of loss of stability were observed^21,22^, as well as the forms of structural failure that are most significant from the point of view of this publication. It was possible to register complex forms of damage, including fractures in the area of composite material plies and delamination.

In addition, the experimental study used a device that records acoustic emission signals, the AMSY-5 system^7,23^. This system is used in non-destructive testing of materials and structures. Acoustic emission (AE) signals are registered when a mechanical wave propagates to the AE sensor. The use of the AMSY-5 AE enables the recording of crack growth sounds, phase transitions, leaks, friction (both external and through crack surfaces), ductility, fibre breakage, debonding, corrosion, wear, capacitive (or partial) discharge, cavitation, impacts, etc. In the tests conducted, the piezoelectric sensor recorded acoustic signals and was mounted directly to the wall of the composite specimen. The sensor-enabled recording test sample parameters include counts, hits, amplitude, energy, and others.

**Fig. 2:**
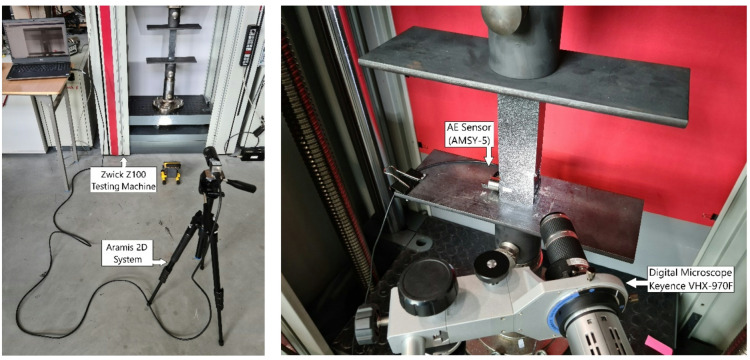
Experimental test stand.

Furthermore, a digital microscope from Keyence model VHX-970 F^24^ with a mobile recording head was also used in the study. The use of the current device enabled, first of all, the real-time recording of failure forms during the experimental tests. It was possible to capture the areas of failure in thin-walled composite structures at a significant zoom level. The above-described test apparatus is shown in Fig. 2.

Experimental tests were conducted on all 36 specimens using a uniform, repeatable procedure. The testing machine heads with flat working surfaces showed very high rigidity, and the flat working surfaces remained parallel to each other throughout the tests. In the central part of the flat working surfaces of the heads, there was a special marking where the test specimens were always mounted axially, thereby significantly eliminating the effect of load misalignment—the research aimed to identify the complex form of failure in composite structures under axial compression. In addition, the impact of the lay-up of composite material on the load-carrying capacity of the structure was analysed, as well as the effect of varying the shape of the cross-section on the behaviour of the structure when it loses its load-carrying capacity.

The above made it possible to evaluate both the failure loads and the failure modes observed at the time of loss of load-carrying capacity of the structure^25–28^. In the present study, the focus is solely on the phenomenon of failure; therefore, attention is given to the failure loads determined experimentally and numerically within the framework of post-buckling equilibrium paths, as well as to the forms of failure and their comparison.

### Finite element method studies

FEA simulations were performed using the finite element method in Abaqus^®^. Proprietary numerical models were developed to faithfully represent parallel experimental studies. FEA models of thin-walled composite structures consisted of 8 plies of material of uniform thickness. The Abaqus software includes a special composite layup editor which provides module to define the plies in the layup. For each ply it is possible to specify the ply’s name, material, thickness, and orientation, as well as the number of integration points and the region of the model to which the ply is assigned. The properties of each ply were modelled individually, which makes it possible to display the results for both the entire structure and the individual composite plies separately. The structure was virtually defined by eight regions of identical thickness constituting individual plies of composite material, with identical parameters specified for each, except for the angle of fibre orientation within the plies. The plies were permanently bonded together (non-separable bond) within the Continuum Shell model. The phenomenon of delamination was not modelled – this will be the subject of future research. In addition, the numerical studies included the design of non-deformable plate elements, enabling the simulation of the compression behavior of the composite structure. Three cases of the structure were modelled, representing the varied closed cross sections of the composite. In addition, during the development of FEM models, four composite lay-ups were considered, in accordance with the previous information provided at the stage of describing the subject of research. In the numerical models, a slight rounding of the vertical edges of the profiles was applied (radius: 0.25 mm) to correctly represent the actual structure.

Discrete models of composite structures were prepared using Continuum Shell SC8R elements (8-node hexahedral continuous stress/displacement general purpose, reduced integration with hourglass control, finite membrane deformation, having 3 translational degrees of freedom per computational node)^11^. The plate elements constituting the supports were prepared using Shell R3D4 elements (a 4-node 2D rigid 3D quadrilateral with 6 degrees of freedom: 3 translational and 3 rotational per computational node)^11^. A mesh density of 2 mm was used for composite structures. For non-deformable plate elements, a mesh density of 2.5 mm was used. A common feature of all numerical models was the maintenance of a constant number of finite elements and computational nodes, since, regardless of the cross-section of the composite structure, the sum of the lengths of its edges was kept constant, with equal height and thickness of the structure. Accordingly, each discrete model consisted of 10,320 finite elements (9200 linear elements of type SC8R and 1120 linear elements of type R3D4) and 19,802 computational nodes. The effect of mesh density on example calculation results was presented in the paper^29^, among others. This approach enabled a reliable comparison of numerical simulation results from independently developed models.

**Fig. 3:**
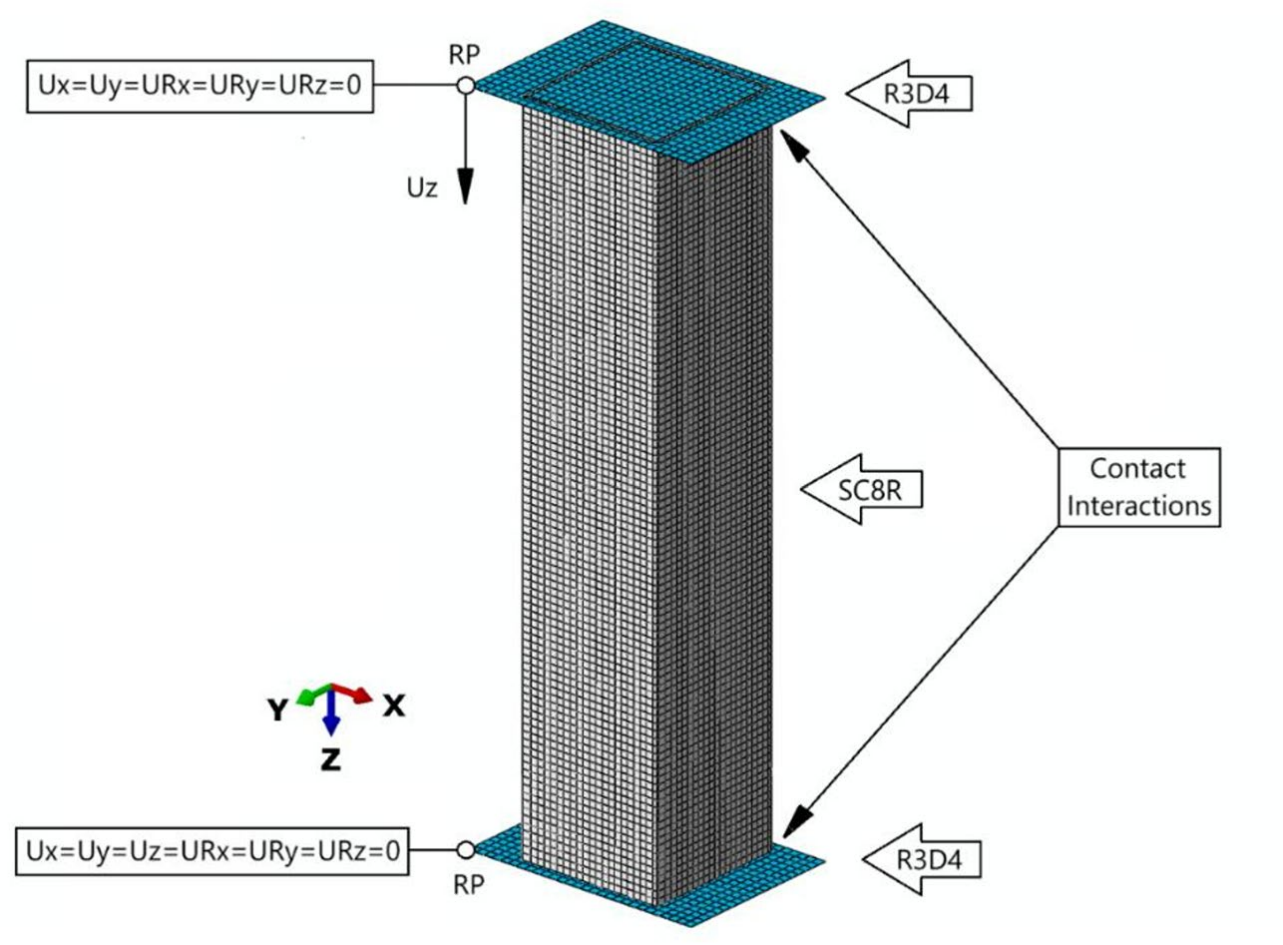
Numerical model with boundary conditions.

In the context of boundary conditions, contact relations with a friction coefficient of 0.2 were used between the composite structure and the supports. In the normal direction, a normal-closed contact type was used, “hard” contact with an option to allow post-contact separation. In Abaqus, “hard” contact refers to the default contact pressure-overclosure relationship where surfaces cannot penetrate each other, meaning they transmit no contact pressure unless in contact and no contact pressure is transmitted across separated surfaces. This approach provides correct interaction between the support elements and the compressed structure. For numerical models, only contact between the end cross-sections of the composite column and non-deformable plates was considered; no additional self-contact was included, as none occurred in the conducted tests. In the performed tests, even before the value of 2.5 mm of structural shortening, a loss of load capacity was observed, with a complex failure mechanism. Of course, if the tests had been conducted for a significantly longer time with higher displacement, resulting in compression, the self-contact interaction would have been considered. Boundary conditions were specified at reference points that were coupled to the elements constituting the supports. This involved a point connected to a top or bottom support, adopting the support’s behaviour— that is, the degrees of freedom at the point were the same as those provided by the support. For the reference point at the bottom support, all degrees of freedom were blocked; for the top support, only one degree of freedom was left free, corresponding to the plate’s displacement along the Z axis. The location of reference points for non-deformable elements is insignificant - because the interaction with non-deformable elements is cumulatively summed and transferred to the reference point. This approach enabled the axial compression process, as a forcing at the top support relative to the Z axis was specified as a displacement, causing compression. The properties of the composite material were defined according to the Lamina-type material model, based on experimentally determined parameters^18^. The numerical model is presented in Fig. 3.

The numerical simulations were carried out by first simulating the linear perturbation —buckle analysis of the structure —determining the buckling mode, which was then sequentially implemented in the nonlinear stability analysis. The problem of linear buckling analysis was presented in detail in the paper^30^. As part of the nonlinear analysis based on the Newton-Raphson method, the loss of load-carrying capacity of the structure was simulated, considering geometric imperfections (0.05 mm of the profiles’ wall thickness, as provided by the WIT-Composites company, which manufactured the profiles).

The present method, which enables nonlinear calculations, enabled the simulation of the loss of load-carrying capacity of the composite structure, with special focus on the complex failure mechanism. The generalized notation of the equation enabling nonlinear calculations follows the form:

1 $$\:{F}^{N}\left({u}^{M}\right)=0$$

Where: *F*^*N*^ constitutes the load component conjugate (to the *N*^*th*^ variable), and *u*^*M*^ is the value of the *M*^*th*^ variable.

The Newton-Raphson method provides a high convergence of the obtained results. After an iteration *i*, an approximation *u*_*i*_^*M*^, to the solution has been obtained. In case that *c*_*i+1*_^*M*^ will be the difference between this solution and the exact solution to the discrete equilibrium equation, then Eq. (1) will take the form:

2 $$\:{F}^{N}({u}_{i}^{M}+{c}_{i+1}^{M})=0$$

Expanding the left side of the above equation, around the approximate solution, the following equation is obtained:

3 $$\:{F}^{N}\left({u}_{i}^{M}\right)+\frac{\partial\:{F}^{N}}{\partial\:{u}^{P}}\left({u}_{i}^{M}\right){c}_{i+1}^{P}+\frac{{\partial\:}^{2}{F}^{N}}{\partial\:{u}^{P}\partial\:{u}^{Q}}\left({u}_{i}^{M}\right){c}_{i+1}^{P}{c}_{i+1}^{Q}+\dots\:=0$$

Moreover, when *u*_*i*_^*M*^ is a close approximation to the solution, the magnitude of each *c*_*i+1*_^*M*^ will be relatively small. Additionally, all components except for the first two terms presented above can be neglected, giving a linear form of equations:

4 $$\:{K}_{i}^{NP}{c}_{i+1}^{P}=-{F}_{i}^{N},\:{\:\:\:K}_{i}^{NP}=\frac{\partial\:{F}^{N}}{\partial\:{u}^{P}}\left({u}_{i}^{M}\right),\:{\:\:\:F}_{i}^{N}={F}^{N}\left({u}_{i}^{M}\right)$$

A further approximation of the solution is as follows:

5 $$\:{u}_{i+1}^{M}={u}_{i}^{M}+{c}_{i+1}^{M}$$

And the iteration continues. The relationships presented above describe the algorithm used for nonlinear calculations.

The nonlinear calculations using the Newton-Raphson method described above were based on a composite material damage model commonly known as progressive failure analysis^31^. This model was based on the fact that damage initiation was governed by the Hashin criterion^32,33^, while damage evolution was governed by the energy criterion^31,34–36^. Damage initiation begins the failure process of the composite material, where, based on the Hashin criterion, it is possible to assess whether the material has initiated the damage process by tension or compression of the fibres, or tension or compression of the matrix:

6 $$\:{F}_{\text{f}}^{\text{t}}=\:{\left(\frac{{\widehat{\sigma\:}}_{11}}{{X}^{\text{T}}}\right)}^{2}+\alpha\:{\left(\frac{{\widehat{\tau\:}}_{12}}{{S}^{\text{L}}}\right)}^{2}=1,\:\text{w}\text{h}\text{e}\text{r}\text{e}\:({\widehat{\sigma\:}}_{11}\ge\:0$$

7 $$\:{F}_{\text{f}}^{\text{c}}=\:{\left(\frac{{\widehat{\sigma\:}}_{11}}{{X}^{\text{c}}}\right)}^{2}=1,\:\text{w}\text{h}\text{e}\text{r}\text{e}\:({\widehat{\sigma\:}}_{11}<0$$

8 $$\:{F}_{\text{m}}^{\text{t}}=\:{\left(\frac{{\widehat{\sigma\:}}_{22}}{{Y}^{\text{T}}}\right)}^{2}+{\left(\frac{{\widehat{\tau\:}}_{12}}{{S}^{\text{L}}}\right)}^{2}=1,\:\text{w}\text{h}\text{e}\text{r}\text{e}\:({\widehat{\sigma\:}}_{22}\ge\:0$$

9 $$\:{F}_{\text{m}}^{\text{c}}=\:{\left(\frac{{\widehat{\sigma\:}}_{22}}{{2S}^{\text{T}}}\right)}^{2}+{\left[{\left(\frac{{Y}^{\text{C}}}{{2S}^{\text{T}}}\right)}^{2}-1\right]\frac{{\widehat{\sigma\:}}_{22}}{{Y}^{\text{C}}}+\left(\frac{{\widehat{\tau\:}}_{12}}{{S}^{\text{L}}}\right)}^{2}=1,\:\text{w}\text{h}\text{e}\text{r}\text{e}\:({\widehat{\sigma\:}}_{22}<0$$

Where: *X*^T^,*X*^C^,*Y*^T^,*Y*^C^,*S*^L^,*S*^T^ are the coefficients of the longitudinal tensile/compressive, transverse tensile/compressive, longitudinal/transverse shear strength; *α* constitutes the contribution of the shear stress; $\:{\widehat{\sigma\:}}_{11},{\widehat{\sigma\:}}_{22,}{\widehat{\tau\:}}_{12,}$ are the components of the effective stress tensor, which is used to evaluate the damage initiation criteria and computed from (10):

10 $$\:\widehat{\sigma\:}=\:M\sigma\:$$

Where: $\:\widehat{\sigma\:}$ is the effective stress tensor, $\:\sigma\:\:$constitutes the (Cauchy) actual stress, *M* is the damage operator.

The damage (matrix) operator has a general notation in the following form:

11 $$\:M=\left[\begin{array}{ccc}\frac{1}{1-{d}_{\text{f}}}&\:0&\:0\\\:0&\:\frac{1}{1-{d}_{\text{m}}}&\:0\\\:0&\:0&\:\frac{1}{1-{d}_{\text{s}}}\end{array}\right]$$

Where: *d*_f_, *d*_m_, *d*_s_ constitute the internal variables of fibre, matrix, as well as shear damage modes, which are derived from damage variables *d*^*t*^_f_, *d*^*c*^_f_, *d*^*t*^_m_, *d*^*c*^_m_, corresponding to the previously mentioned four modes, as follows:

12 $$\:{d}_{\text{f}}=\left\{\begin{array}{c}{d}_{\text{f}}^{\text{t}},\:\:\:\:if\:\:\:{\widehat{\sigma\:}}_{11}\ge\:0\\\:{d}_{\text{f}}^{\text{c}},\:\:\:\:if\:\:\:{\widehat{\sigma\:}}_{11}<0\end{array}\right.$$

13 $$\:{\:\:d}_{\text{m}}=\left\{\begin{array}{c}{d}_{\text{m}}^{\text{t}},\:\:\:\:if\:\:\:{\widehat{\sigma\:}}_{22}\ge\:0\\\:{d}_{\text{m}}^{\text{c}},\:\:\:\:if\:\:\:{\widehat{\sigma\:}}_{22}<0\end{array}\right.$$

14 $$\:{\:\:d}_{\text{s}}=1-\left(1-{d}_{f}^{t}\right)\left(1-{d}_{\text{f}}^{\text{c}}\right)\left(1-{d}_{\text{m}}^{\text{t}}\right)\left(1-{d}_{\text{m}}^{\text{c}}\right)$$

Before the damage initiation and evolution, the damage operator *M* is equal to the identity matrix, therefore $\:\widehat{\sigma\:}=\sigma\:$. Once the damage initiation and evolution phenomena occur for at least one mode, the damage operator becomes significant in the damage initiation criteria of other modes. In the paper above, damage initiation in fibre-reinforced composites was discussed. A more significant phenomenon from the point of view of analysing the loss of structural capacity is damage evolution, the consequence of damage initiation. Once a damage initiation phenomenon is fulfilled, further loading will cause a reduction (degradation) of material stiffness coefficients. Before damage initiation, the material is linearly elastic and obeys the stiffness matrix of a plane-stress orthotropic material. The material’s response is then calculated based on:

15 $$\:\sigma\:=\:{C}_{d}\epsilon\:$$

where *ε* is the strain and *C*_d_ is the damaged elasticity matrix, which has the form:

16 $$\:{C}_{d}=\frac{1}{D}\left[\begin{array}{ccc}{\left(1-{d}_{f}\right)E}_{1}&\:\left(1-{d}_{f}\right){\left(1-{d}_{m}\right)\nu\:}_{21}{E}_{1}&\:0\\\:\left(1-{d}_{f}\right)\left(1-{d}_{m}\right){\nu\:}_{12}{E}_{2}&\:{\left(1-{d}_{m}\right)E}_{2}&\:0\\\:0&\:0&\:D\left(1-{d}_{s}\right)G\end{array}\right]$$

Where:

17 $$\:D=1-{v}_{12}{v}_{21}\left(1-{d}_{\text{f}}\right)\left(1-{d}_{\text{m}}\right)$$

In the case of the above Eqs. (16–17), *d*_f_, *d*_m,_ and *d*_s_ represent the current state of fibre/matrix/shear damage, *E*_1_ is the Young’s modulus in the fibre direction, *E*_2_ is the Young’s modulus in the matrix direction, *G* is the shear modulus, *v*_12_ and *v*_21_ are Poisson’s ratios.

The damage evolution law is based on the fracture energy released during the damage process, *G*_*c*_. Regarding the above, it is necessary to specify energy parameters *G*^c^_ft_ = 133 N/mm, *G*^c^_fc_ = 40 N/mm, *G*^c^_mt_ = 0.6 N/mm, *G*^c^_mc_ = 2.1 N/mm (value of energies dissipated during damage for fibre tension/compression, as well as matrix tension/compression)^11^. The damage parameters are described in detail in a paper presenting the fundamental principles of damage variables^31^. This paper describes the principles of modelling damage based on the damage variables specified within this paper, which is related to the Hashin criterion. The energy parameters were adopted from an earlier paper^11^.

## Research results

Based on the research carried out, the focus was on determining post-buckling equilibrium paths for composite profiles with closed sections, from which the failure loads were determined. In addition, the failure modes representing the composite failure mechanism of the composite material were determined. The above was determined for both experimental tests and numerical simulations using FEM. This paper focuses solely on the loss of load-carrying capacity in composite structures. Previous publications have presented critical (buckling) load values that are significant in the context of structural stability^29,37^.

**Fig. 4:**
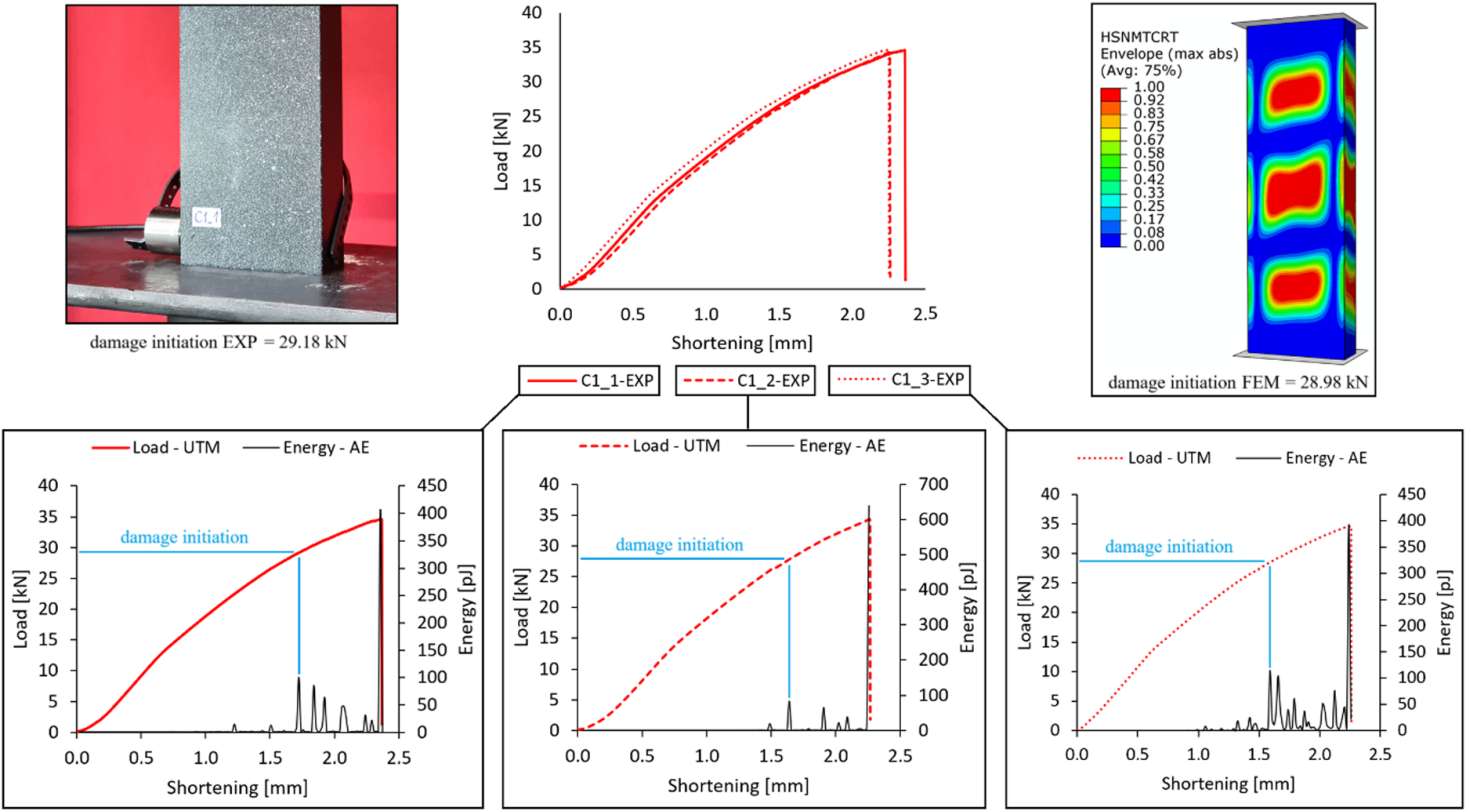
Damage initiation on the example of C1-type profiles.

Initially, exemplary results from the acoustic emission method for evaluating damage initiation in composite materials were presented. The research results were presented using actual specimens of type C1 (a profile with a cross-sectional area of 60 × 20 mm and a composite lay-up [0°/45°/-45°/90°]) – Fig. 4. Generally, an energy (or amplitude) signal was used, which, in most cases, enabled estimation of the loads that initiated the damage.

In the case of acoustic emission used to assess damage initiation, it was possible to estimate the loads that initiate damage in composite structures. Of several possible acoustic emission signals for analysis (number of counts, hits, energy, and amplitude), the energy signal and, in some cases, the amplitude signal were most commonly used to evaluate damage initiation. Damage initiation began with the first significant peak of the acoustic emission signal, where, for energy, it was about 100 pJ, as shown in Fig. 4. In numerical simulations, damage initiation was evaluated using the Hashin criterion; in all cases studied, damage was initiated due to matrix tension in the composite material, parameter HSNMTCRT. Table 2 summarizes the load values at which damage initiation occurred.

**Table 2:** Experimental-numerical values of damage initiation loads.

Spec. Type	Spec. No.						
	1	2	3	EXP_avg_	FEM	FEM/EXP_avg_	FEM/EXP_avg_ (mean) (variance)
A1	28.38 kN	26.98 kN	28.46 kN	27.94 kN	29.03 kN	1.04	(1.05) (0.0017)
B1	29.49 kN	29.35 kN	28.41 kN	29.08 kN	30.03 kN	1.03	
C1	29.18 kN	27.74 kN	28.50 kN	28.47 kN	28.98 kN	1.02	
A2	20.23 kN	23.56 kN	21.04 kN	21.61 kN	24.69 kN	1.14	
B2	27.54 kN	26.92 kN	27.62 kN	27.36 kN	28.68 kN	1.05	
C2	31.08 kN	30.13 kN	30.30 kN	30.50 kN	31.07 kN	1.02	
A3	27.93 kN	33.01 kN	29.64 kN	30.19 kN	33.95 kN	1.12	
B3	33.89 kN	34.27 kN	33.96 kN	34.04 kN	36.02 kN	1.06	
C3	26.65 kN	25.90 kN	27.73 kN	26.76 kN	27.10 kN	1.01	
A4	22.90 kN	25.56 kN	21.52 kN	23.33 kN	24.65 kN	1.06	
B4	24.96 kN	25.53 kN	26.68 kN	25.72 kN	25.75 kN	1.00	
C4	23.90 kN	23.06 kN	21.16 kN	22.71 kN	24.58 kN	1.08	

In the present study, damage initiation was not described in detail because it had been the subject of previous studies^25^.

**Fig. 5:**
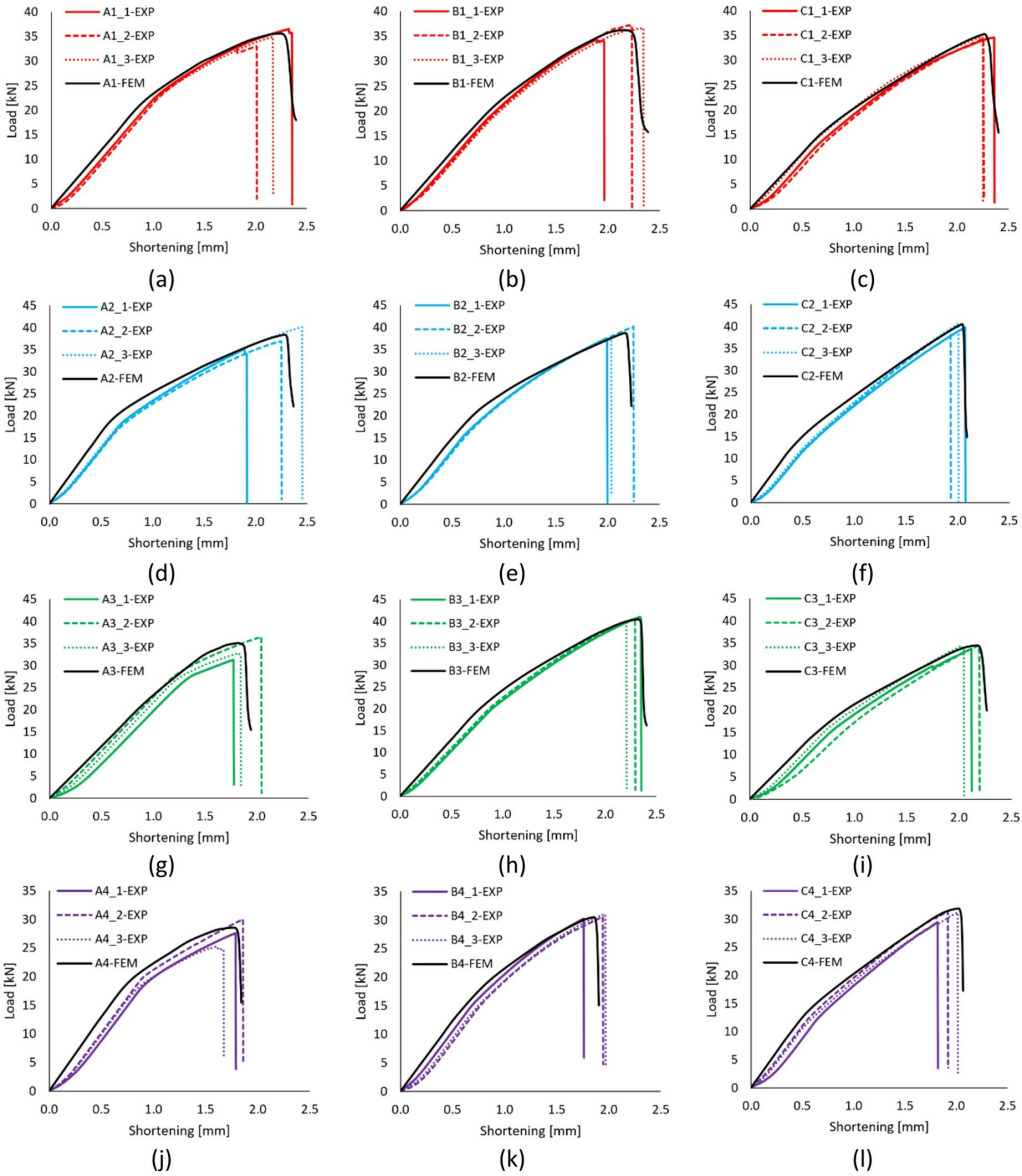
Experimentally determined post-buckling characteristics: (**a**) A1-type, (**b**) B1-type, (**c**) C1-type, (**d**) A2-type, (**e**) B2-type, (**f**) C2-type, (**g**) A3-type, (**h**) B3-type, (**i**) C3-type, (**j**) A4-type, (**k**) B4-type, (**g**) C4-type.

Post-buckling equilibrium paths of the structure are presented for all profiles, including those with various cross-sections and different lay-ups of the composite material. The characteristics mentioned earlier are presented in Fig. 5.

The characteristic inflection points on the curves (stiffness changes) were usually associated with a loss of stability, as reported in the publication^29^. Experimental post-buckling equilibrium paths showed high stiffness from the outset due to the compact construction, in contrast to previously commonly studied profiles with open Sects^1,11^. The experimental load-shortening curves shown in each graph followed a similar course for the three specimens in each profile type. The above indicates the high quality of the manufactured profiles, as evidenced by the high repeatability of the experimental curves. The curves obtained from numerical simulations were very similar to the experimental curves. The experimentally determined characteristics enabled the determination of the failure loads. The failure load values have been presented collectively in Table 3 to quantify the loss of load-carrying capacity better.

**Table 3:** Experimental-numerical values of failure loads.

Spec. Type	Spec. No.						
	1	2	3	EXP_avg_	FEM	FEM/EXP_avg_	FEM/EXP_avg_ (mean) (variance)
A1	36.51 kN	33.01 kN	34.92 kN	34.81 kN	35.59 kN	1.02	(1.02) (0.0002)
B1	34.29 kN	37.20 kN	36.65 kN	36.05 kN	36.21 kN	1.00	
C1	34.62 kN	34.32 kN	34.72 kN	34.55 kN	35.26 kN	1.02	
A2	35.05 kN	36.90 kN	40.12 kN	37.36 kN	38.36 kN	1.03	
B2	37.51 kN	40.14 kN	37.72 kN	38.46 kN	38.70 kN	1.01	
C2	39.80 kN	39.15 kN	40.57 kN	39.84 kN	40.46 kN	1.02	
A3	31.29 kN	36.44 kN	32.79 kN	33.51 kN	35.06 kN	1.05	
B3	41.03 kN	40.67 kN	39.79 kN	40.50 kN	40.62 kN	1.00	
C3	33.66 kN	34.42 kN	34.37 kN	34.15 kN	34.46 kN	1.01	
A4	27.58 kN	30.02 kN	25.26 kN	27.62 kN	28.54 kN	1.03	
B4	30.24 kN	30.57 kN	30.95 kN	30.58 kN	30.69 kN	1.00	
C4	29.45 kN	31.42 kN	31.17 kN	30.68 kN	31.89 kN	1.04	

The main objective of the present study was to determine the limit loads of composite specimens with four different lay-ups and three different cross-sections. Since a total of 36 specimens were tested in accordance with the information presented in the study section, the load-carrying capacity of thin-walled composite structures could be evaluated. This evaluation was carried out in terms of both the influence of the lay-up of the composite plies and the impact of the cross-section shape on the load-carrying capacity of the structure.

Based on the obtained post-buckling equilibrium paths, some correlations were observed. It was observed that regardless of the type of cross-section, as well as the arrangement of laminate plies, composite profiles with closed cross-sections lost their load-carrying capacity when the specimen was shortened approximately 2 mm. The highest average failure load in the experimental tests was observed for the B3 profile (P_f_ = 40.5 kN), while the lowest average failure load was observed for the A4 profile (P_f_ = 27.62 kN). Consequently, based on the comparison of the average failure loads (from the maximum to the minimum), it was estimated that the B3-type profile had a 1.47 times higher load capacity than the A4-type profile. In numerical simulations, a similar situation occurred: for the B3 type profile, the failure load P_f_ = 40.62 kN, and for the A4 type profile, P_f_ = 28.54 kN. The above indicates that the B3-type profile had a 1.42 times higher load capacity than the A4-type profile. The ratio between the failure load values obtained by numerical simulations and the average failure load values from experimental tests ranged from 1.00 to 1.05. The maximum discrepancy between the failure load obtained by numerical simulation and the average experimentally determined failure load occurred for the A3 type profile, with a ratio of 1.05. The minimal differences between the failure loads obtained by numerical simulations and the average values from experimental tests indicate the high quality of the numerical models developed.

Based on the results, the effect of the cross-section shape on the load capacity for a particular lay-up was also observed. For example, for the first type of lay-up, 1 - [0°/45°/-45°/90°]_s_, it was noted that the cross-section shape has no significant effect on the failure values. It was observed that the average experimental failure load for the B1-type profile was only 1.04 times that of the C1-type profile. In other cases, the discrepancies in the average failure loads for a particular lay-up across different cross sections were slightly higher. In the case of the second lay-up of the composite material 2 - [0°/90°/0°/90°]_s_, it was observed that the average value of the failure load for the C2 profile relative to the A2 profile was 1.07 times higher. For the third lay-up 3 - [45°/-45°/90°/0°]_s_, it was noted that the average value of the failure load for profile B3 relative to profile A3 was 1.21 times higher, while for the fourth and also the last lay-up 4 - [90°/-45°/45°/0°]_s_, the average value of the failure load for profile C4 relative to profile A4 was 1.11 times higher. The most significant discrepancy between the average failure loads for various profile cross sections occurred for the third lay-up [45°/-45°/90°/0°]_s_. This indicates that, regardless of the identical sums of the lengths of the edges of each cross-section, changing the ratio of the lengths of the opposite edges, for some lay-ups, has a negligible effect on the failure loads, whereas for others it has a greater effect.

**Fig. 6:**
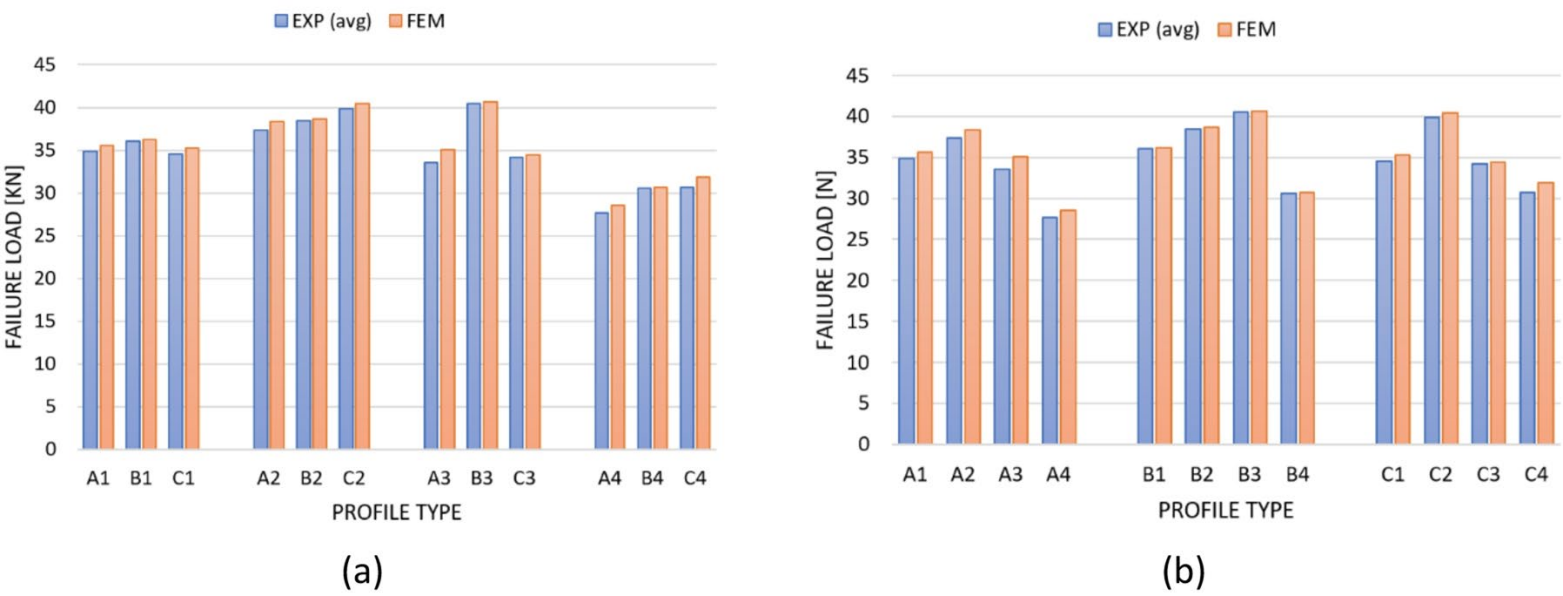
Summary of failure loads in graphical form: (**a**) the impact of cross-section shape on load carrying capacity for a particular type of composite lay-up, (**b**) the impact of the composite lay-up on the load carrying capacity for a particular type of cross-section.

Based on the conducted tests, it was possible to compare the values of failure loads with the demonstration of the apparent influence of the shape of the cross-section for a particular type of composite lay-ups, as well as the influence of the composite lay-ups for a specific type of cross-section on the load-carrying capacity of the structure. The above allowed us to demonstrate whether the cross-section shape or the composite lay-up has a greater influence on the load-carrying capacity of thin-walled composite structures. Figure 6 shows the aforementioned issues: Fig. 6a presents the effect of cross-section shape, and Fig. 6b shows the impact of composite lay-up on the load-carrying capacity of the structure.

Based on the conducted tests (comparing the average values from experimental tests and FEM results), it was found that the composite lay-up, rather than the cross-section shape, has a significantly greater influence on the load-carrying capacity of the structure for the analysed profiles. Using the results of Fig. 6a as an example, it can be observed that for most of the lay-ups of the composite material i.e. 1, 2 and 4 the influence of the cross-section (A, B and C) was not significant. Only for the third composite lay-up [45°/-45°/90°/0°]_s_ it was observed that the influence was more important. More significant, however, in terms of load-carrying capacity, was the effect of the composite lay-up on the load-carrying capacity of thin-walled composite profiles, as already mentioned. In the case of profiles with the first type of cross-section (type A), it was noted that the second composite lay-up [0°/90°/0°/90°]_s_ relative to the fourth composite lay-up [90°/-45°/45°/0°]_s_ showed a 1.35 times higher load capacity. For profiles with the second type of cross-section (type B), it was observed that the third composite lay-up [45°/-45°/90°/0°]_s_ relative to the fourth composite lay-up [90°/-45°/45°/0°] showed 1.32 times higher load capacity. For profiles with the third type of cross-section (type C), it was demonstrated that the second composite lay-up [0°/90°/0°/90°]_s_ relative to the fourth composite lay-up [90°/-45°/45°/0°], showed 1.3 times higher load capacity.

Consequently, it was noted that composite profiles with the fourth composite lay-up showed the lowest load capacity, while those with the second and third composite lay-ups showed the highest load capacity. Based on the experimental results, the effect of the cross-section shape had a lower impact (B3/A3 = 1.21) on the load-carrying capacity of the structure than the composite lay-up (A2/A4 = 1.35). The aforementioned difference may result from geometric imperfections that were difficult to evaluate during the manufacturing of thin-walled composite profiles. Certain geometric imperfections are unavoidable, and most often lead to some discrepancies in the test results.

**Fig. 7:**
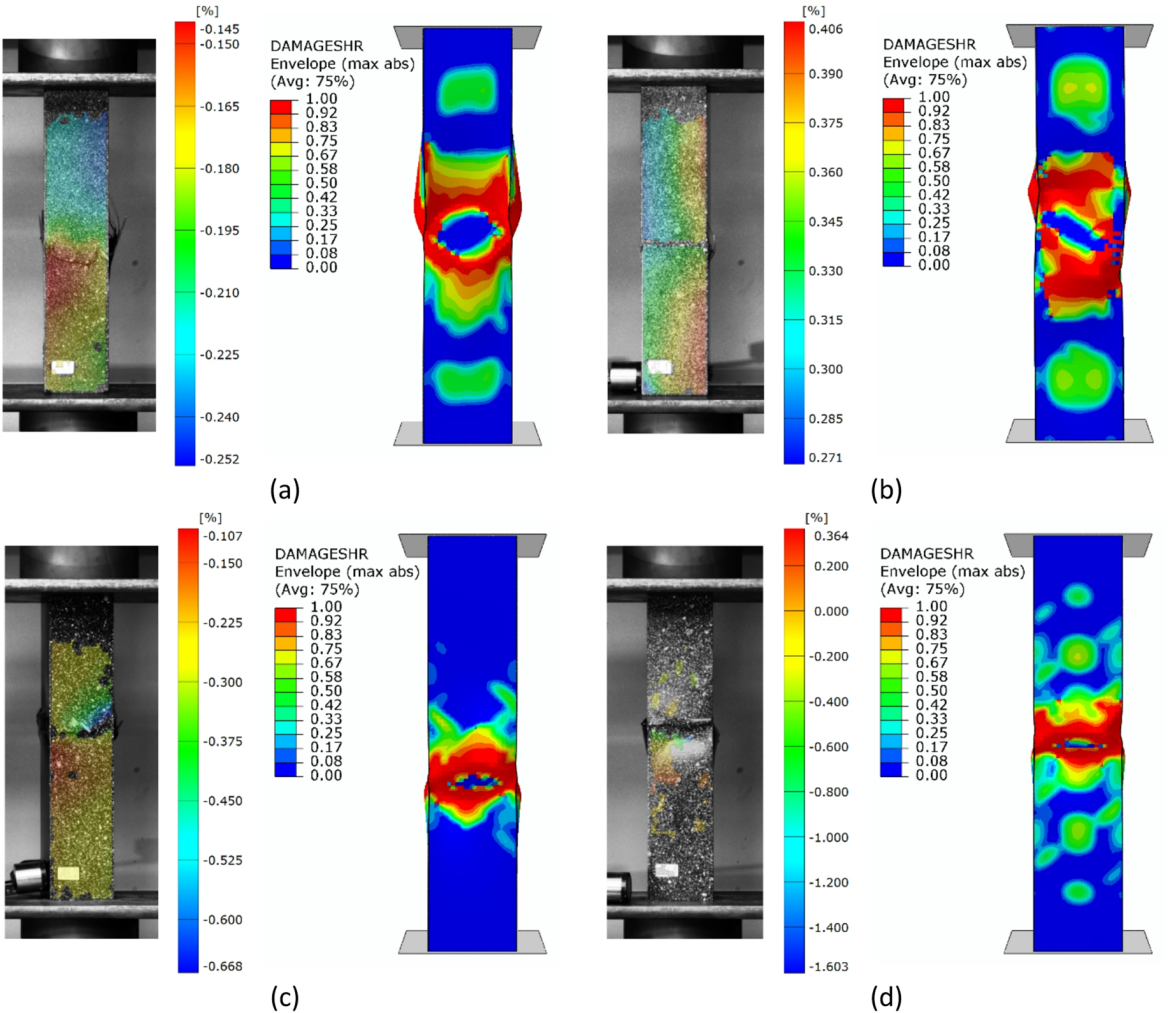
Experimental - numerical comparison of the failure forms on the example of profiles with cross-section of type A: (**a**) lay-up of type 1, (**b**) lay-up of type 2, (**c**) lay-up of type 3, (**d**) lay-up of type 4.

**Fig. 8:**
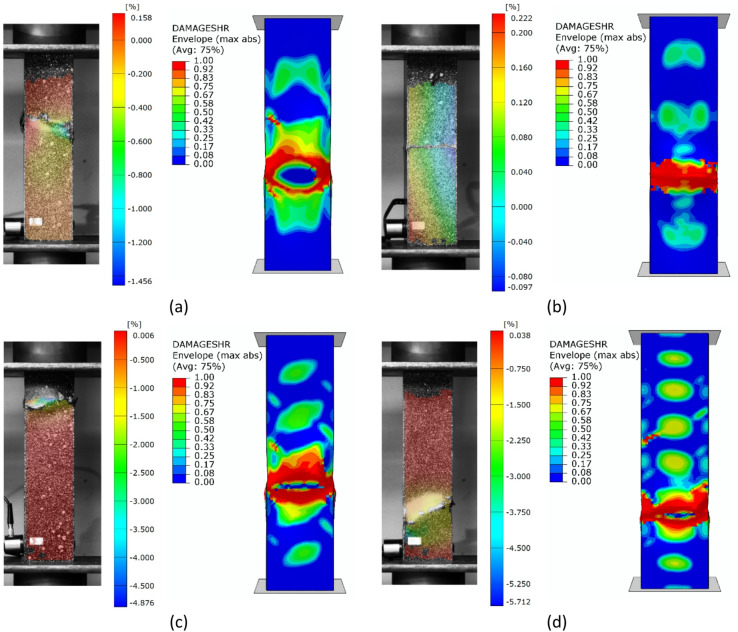
Experimental - numerical comparison of the failure forms on the example of profiles with cross-section of type B: (**a**) lay-up of type 1, (**b**) lay-up of type 2, (**c**) lay-up of type 3, (**d**) lay-up of type 4.

**Fig. 9:**
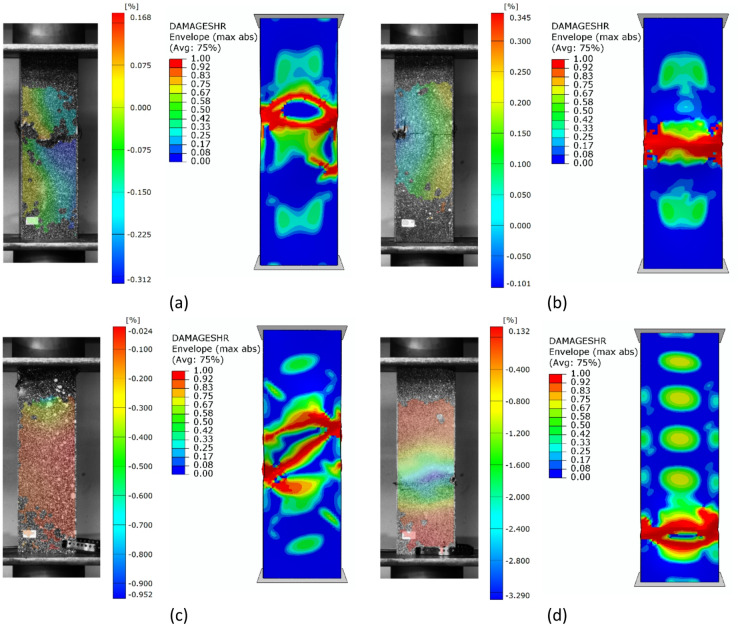
Experimental - numerical comparison of the failure forms on the example of profiles with cross-section of type C: (**a**) lay-up of type 1, (**b**) lay-up of type 2, (**c**) lay-up of type 3, (**d**) lay-up of type 4.

Regarding the parallel experimental and numerical studies, it was decided to provide a summary of the forms of structural failure. To better visualize the failure phenomenon, the forms of damage were compared using an example of selected profiles from experimental tests and FEM simulation results - as shown in Figs. 7, 8 and 9. The following figures present the failure forms obtained during experimental testing, which were additionally captured using an optical system for measuring deformation, as well as the failure forms from the progressive failure analysis conducted using FEM. In the graphical presentation of results using the Aramis 2D system, the deformation (strain) parameter in the longitudinal (vertical) direction of the column was used. As part of the test result processing, a special spatial median filter was applied to highlight longitudinal deformations. During the study, the 3D Aramis system was unavailable (which would have been appropriate for this type of analysis; in future studies, this type of testing equipment will be used). In some cases, the location of the damage did not fully correspond between experimental tests and numerical simulations. This could have been due to the fact that some profiles had slightly different geometric imperfections at the manufacturing stage, which could have had a significant impact on the location of the damage. The damage in specimens from the same set (3 specimens from the same series) was similar in nature and location.

**Fig. 10:**
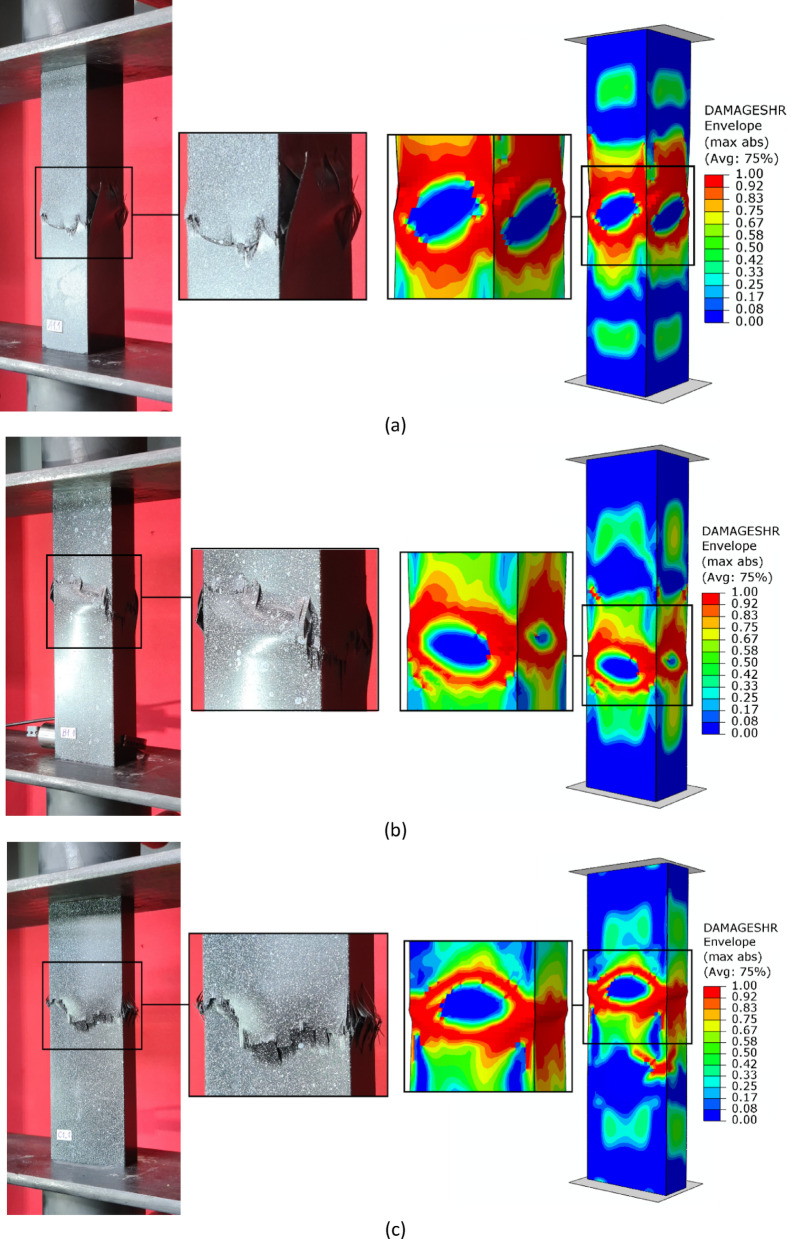
Comparison of experimental and numerical failure forms on the example of selected profiles: (**a**) cross-section type A, (**b**) cross-section type B, (**c**) cross-section type C.

Based on the study, some similarities were observed in the results of experimental studies and numerical simulations. It was observed that, regardless of composite lay-up and cross-section shape, most composite columns exhibited a dominant failure mode in the region corresponding to half the height of the composite profiles. The above was observed both for the example experimental specimens shown in Figs. 7, 8 and 9, as well as for the numerical simulations. In the experimental testing, the results were presented using an optical deformation measurement system. In contrast, in the case of numerical simulations, the results were shown based on the selected DAMAGESHR parameter within the framework of progressive failure analysis (which considered damage components from fibre and matrix failure due to compression and tension). In the numerically analysed cases, the damage began with the fulfilment of a parameter related to the matrix tension of the composite material (DAMAGEMT), and it was this component that was the direct cause of the onset and further progression of the damage process. Furthermore, to better qualitatively compare the obtained failure forms, it was decided to present the composite failure forms for each type of cross-section, with detailed representations of the failure areas – Fig. 10.

The shapes of the failure areas were very similar in both experimental studies and numerical simulations. In experimental studies, both fracture of composite material plies and delamination phenomena were observed within the composite failure mechanism, which will be the subject of further numerical simulations based on future studies that include failure models such as XFEM and CZM. Future research activities will be carried out based on the above and other advanced failure models, as outlined in scientific publications^6,11^.

**Fig. 11:**
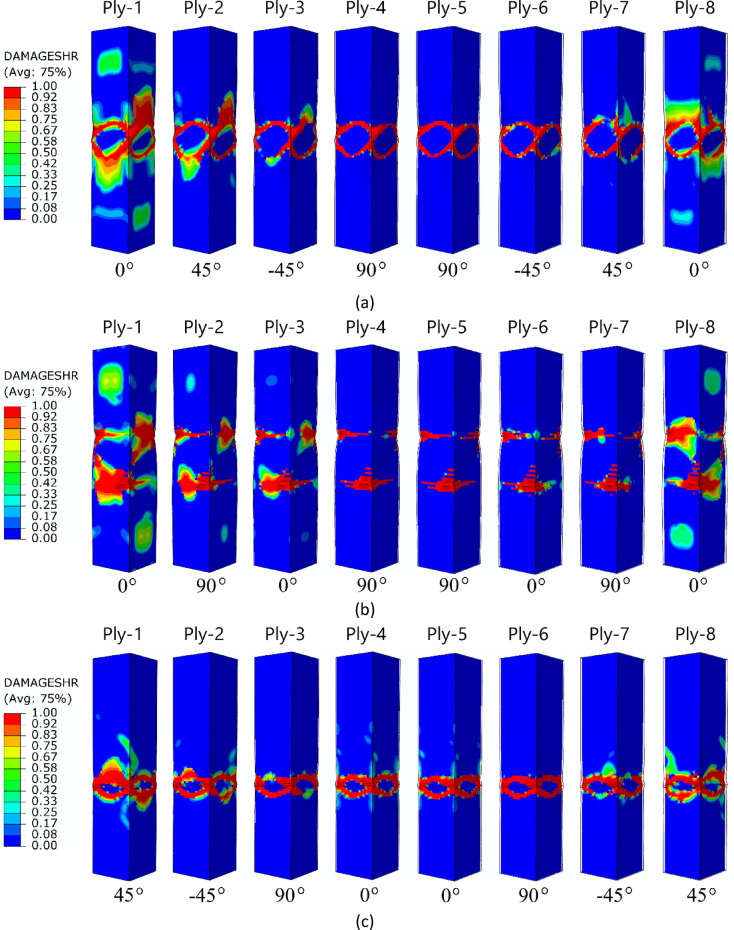

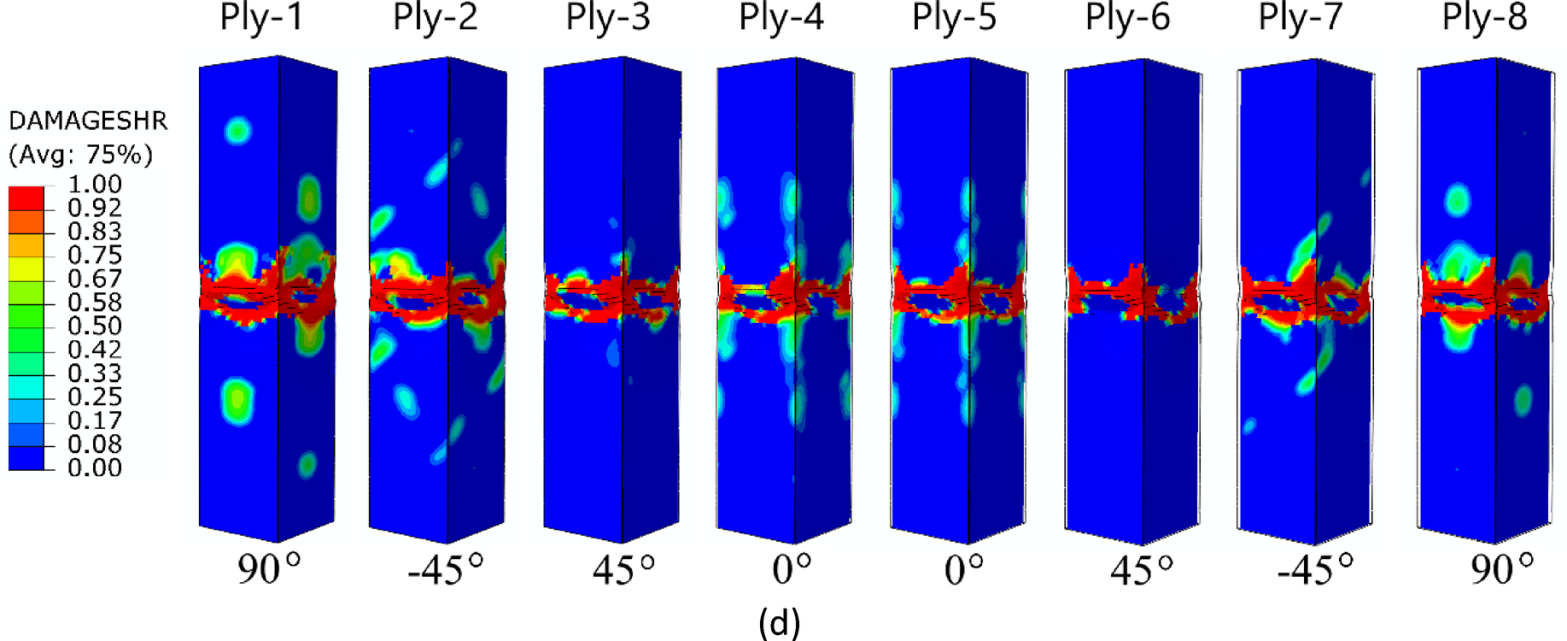
The failure phenomenon presented for each ply of the composite material (FEM): (**a**) composite type A1, (**b**) composite type A2, (**c**) composite type A3, (**d**) composite type A4.

For an in-depth analysis of the failure phenomenon in a qualitative context, a graphical representation of the failure of individual plies in a composite material, for an A-type cross-section, is shown in Fig. 11. Based on the numerical simulations performed, among other things, the effect of the lay-up of the composite material (1,2,3, and 4 composite lay-ups) on the failure of all the plies of which each composite structure consisted was presented. This enabled a more precise visualization of the complex nature of failure in thin-walled composite profiles with closed cross-sections. The failure forms of each ply were presented using the most representative DAMAGESHR parameter as an example.

**Fig. 12:**
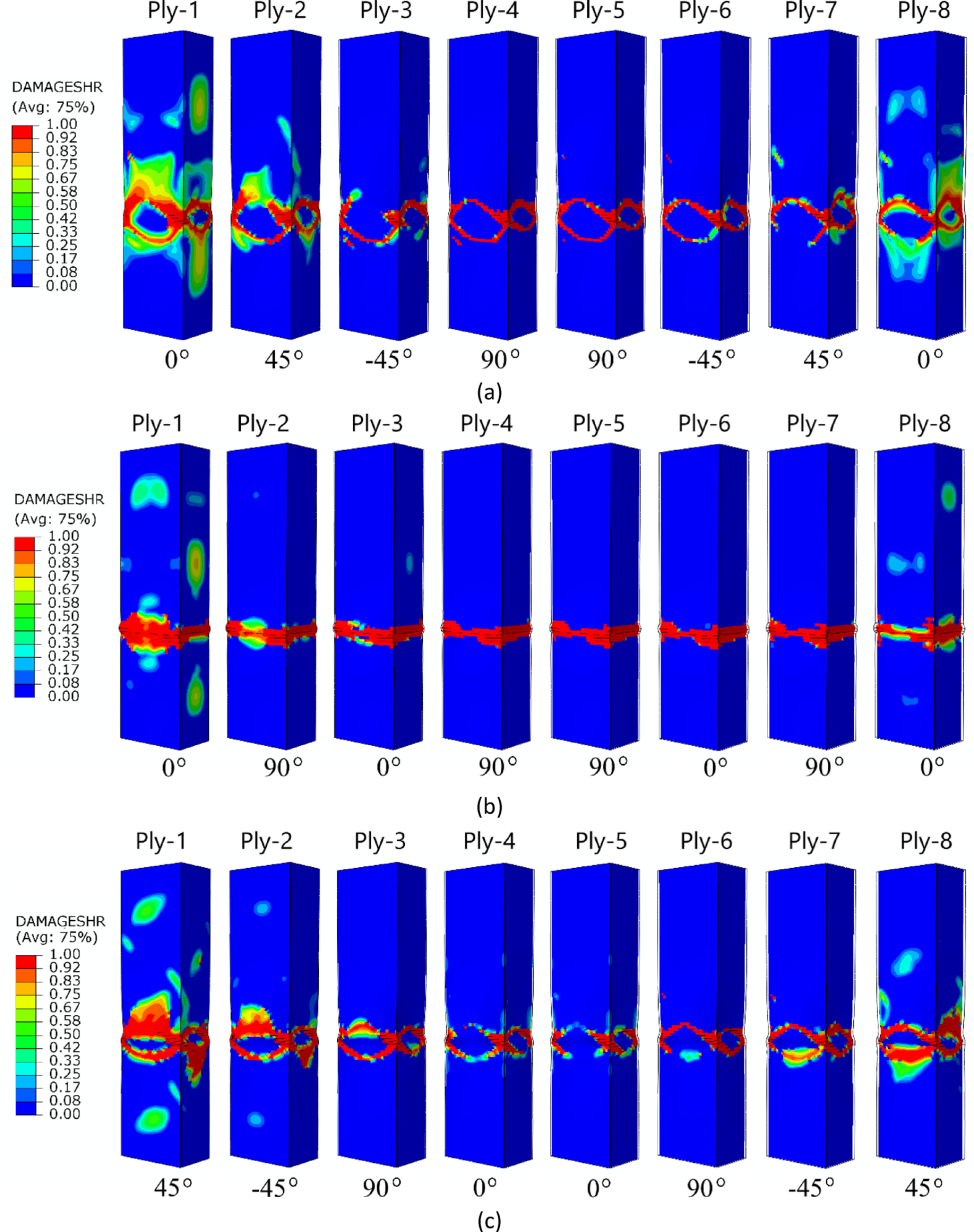

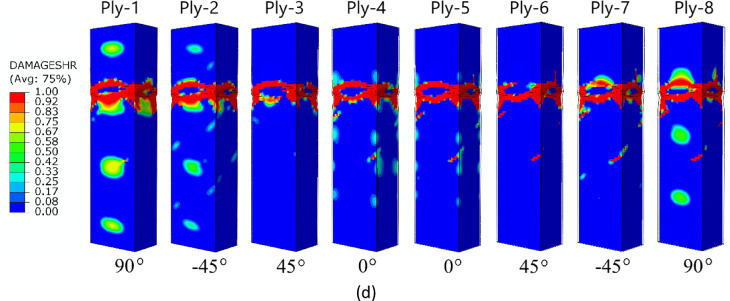
The failure phenomenon presented for each ply of the composite material (FEM): (**a**) composite type B1, (**b**) composite type B2, (**c**) composite type B3, (**d**) composite type B4.

**Fig. 13:**
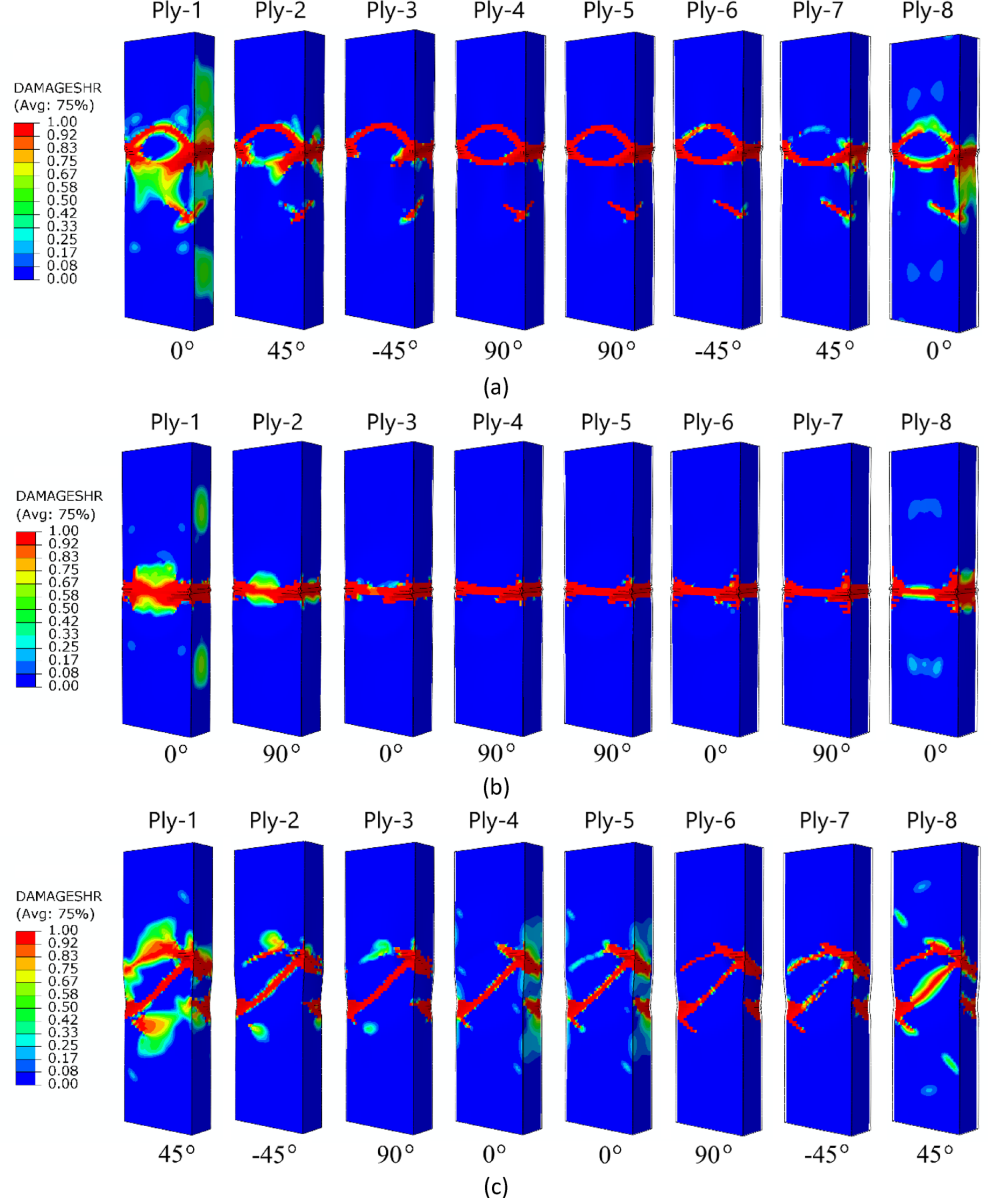

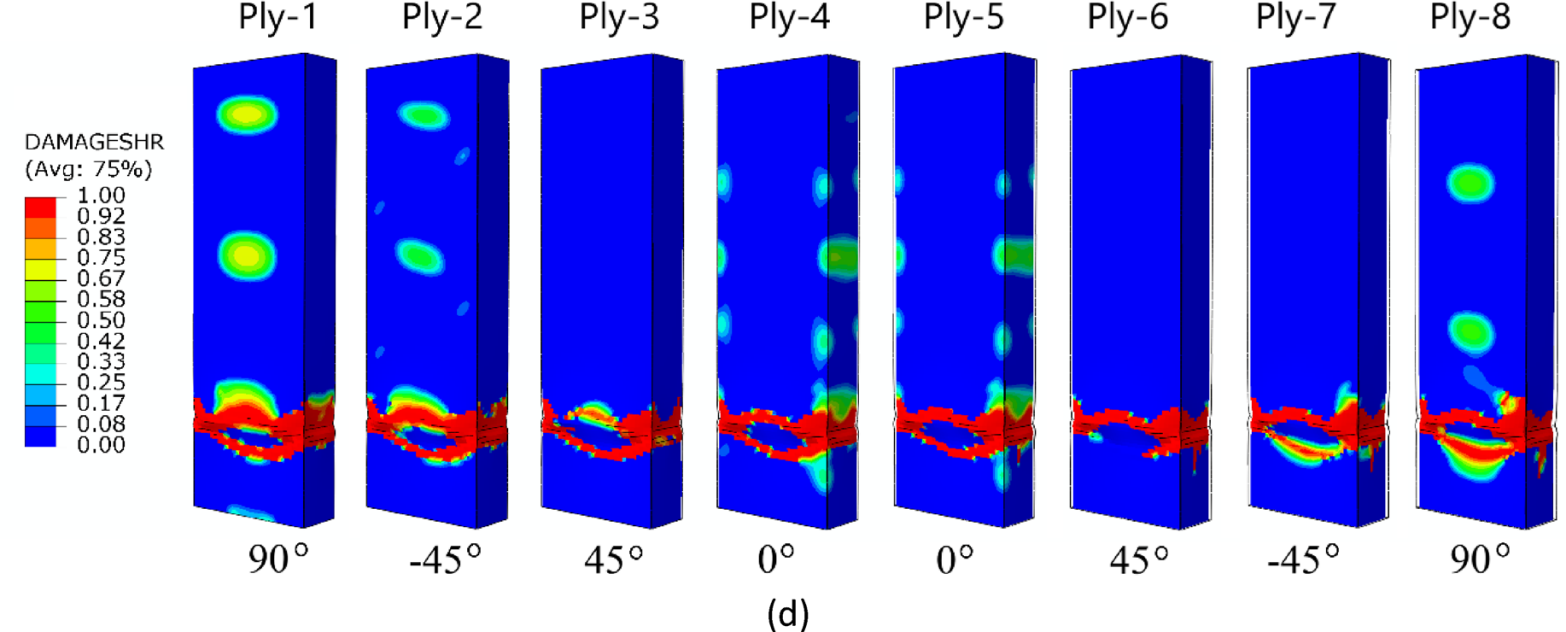
The failure phenomenon presented for each ply of the composite material (FEM): (**a**) composite type C1, (**b**) composite type C2, (**c**) composite type C3, (**d**) composite type C4.

The failure forms shown in Fig. 11 (A-type cross-section profile) enabled observation of the level of damage in each ply of the composite structure. In all cases analysed by numerical simulations, it was observed that, regardless of the composite lay-up, the most extensive failure area occurred in the extreme (outermost) plies of the composite (ply 1 and ply 8). The inner plies of the composite material showed significantly lower failure areas, indicating that the “core” in the form of the inner plies provided high strength to the composite. A similar situation occurred for the other two types of composite material cross-sections (type B and C) – Figs. 12 and 13. In all cases, it was observed that the dominant forms of damage occurred in the outermost plies of the composite material - regardless of the shape of the cross-section and the composite lay-up.

**Fig. 14:**
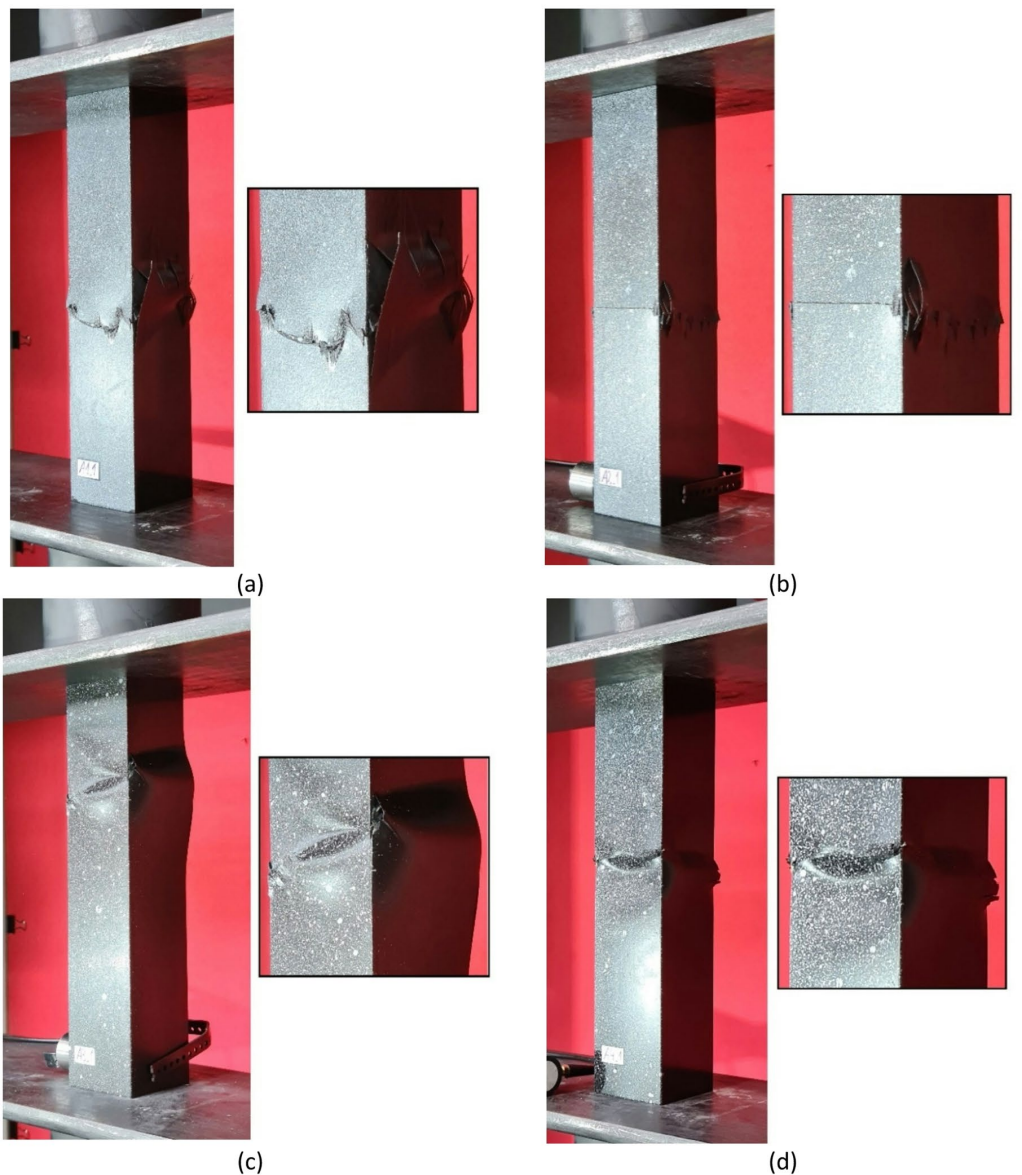
The failure phenomenon presented for each ply of the composite material (EXP): (**a**) composite type A1, (**b**) composite type A2, (**c**) composite type A3, (**d**) composite type A4.

The extreme (outermost) plies were the most susceptible to failure, as confirmed by the experimental test results shown in Fig. 14.

The failure forms presented in Fig. 14 confirmed that the failure was mainly visible on the external plies of the composite material. During the experimental study, it was noted that the external plies of the material for the example profile with a cross-section of type A were damaged by both fracture and delamination, confirming the complex mechanism of failure of the composite material. Therefore, the subject of future research will be the simulation of the aforementioned failure forms using advanced numerical models.

**Fig. 15:**
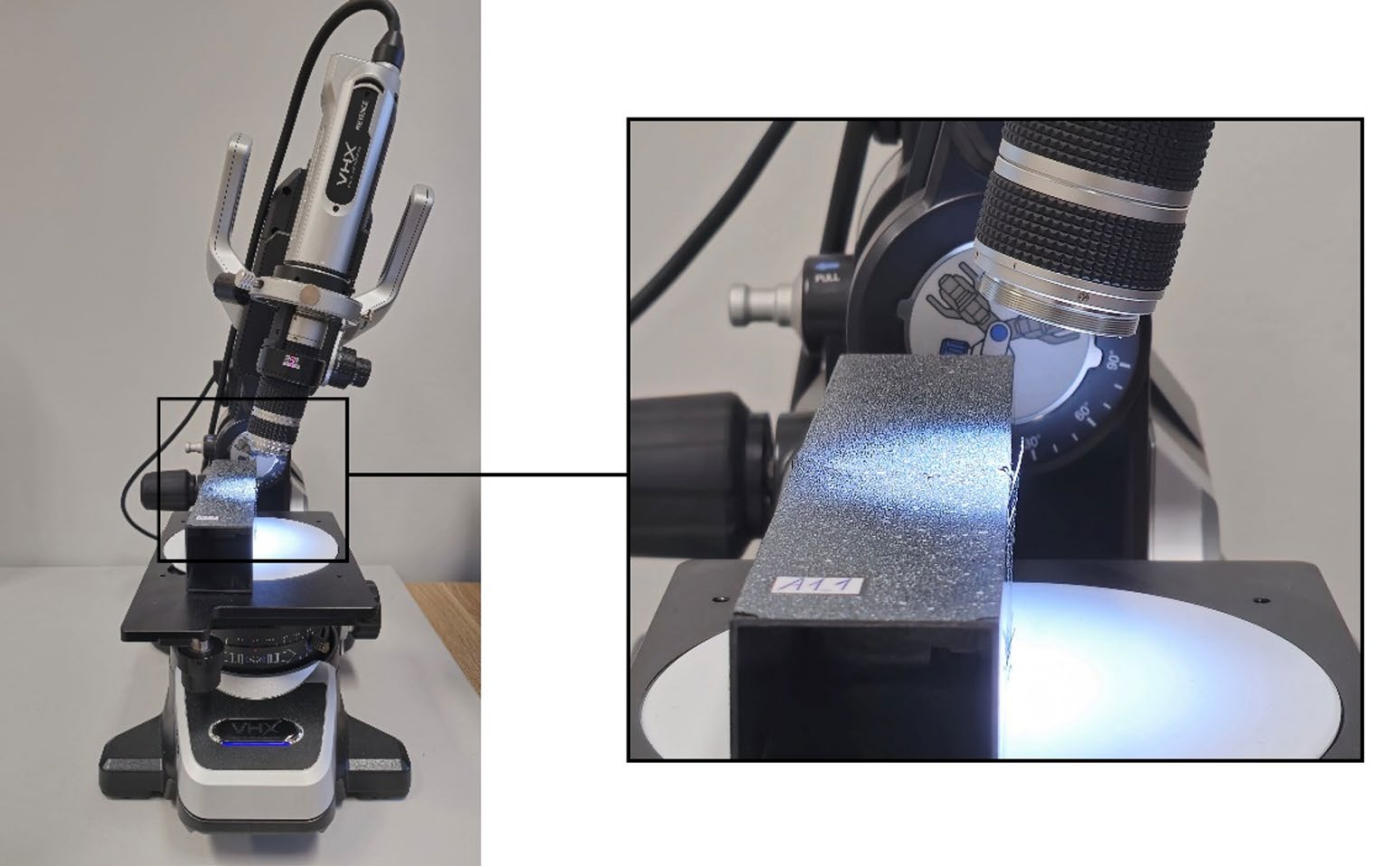
Observation of the failure area using the varying tilt angle of the microscope head.

**Fig. 16:**
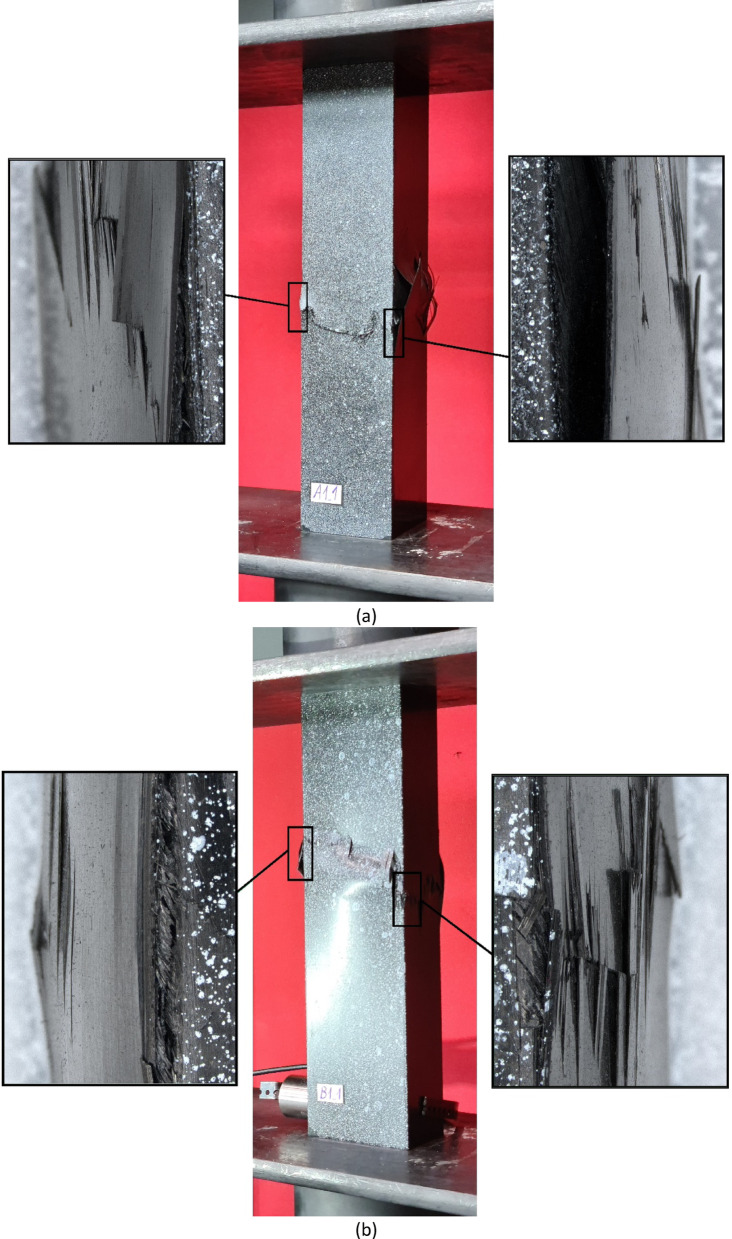

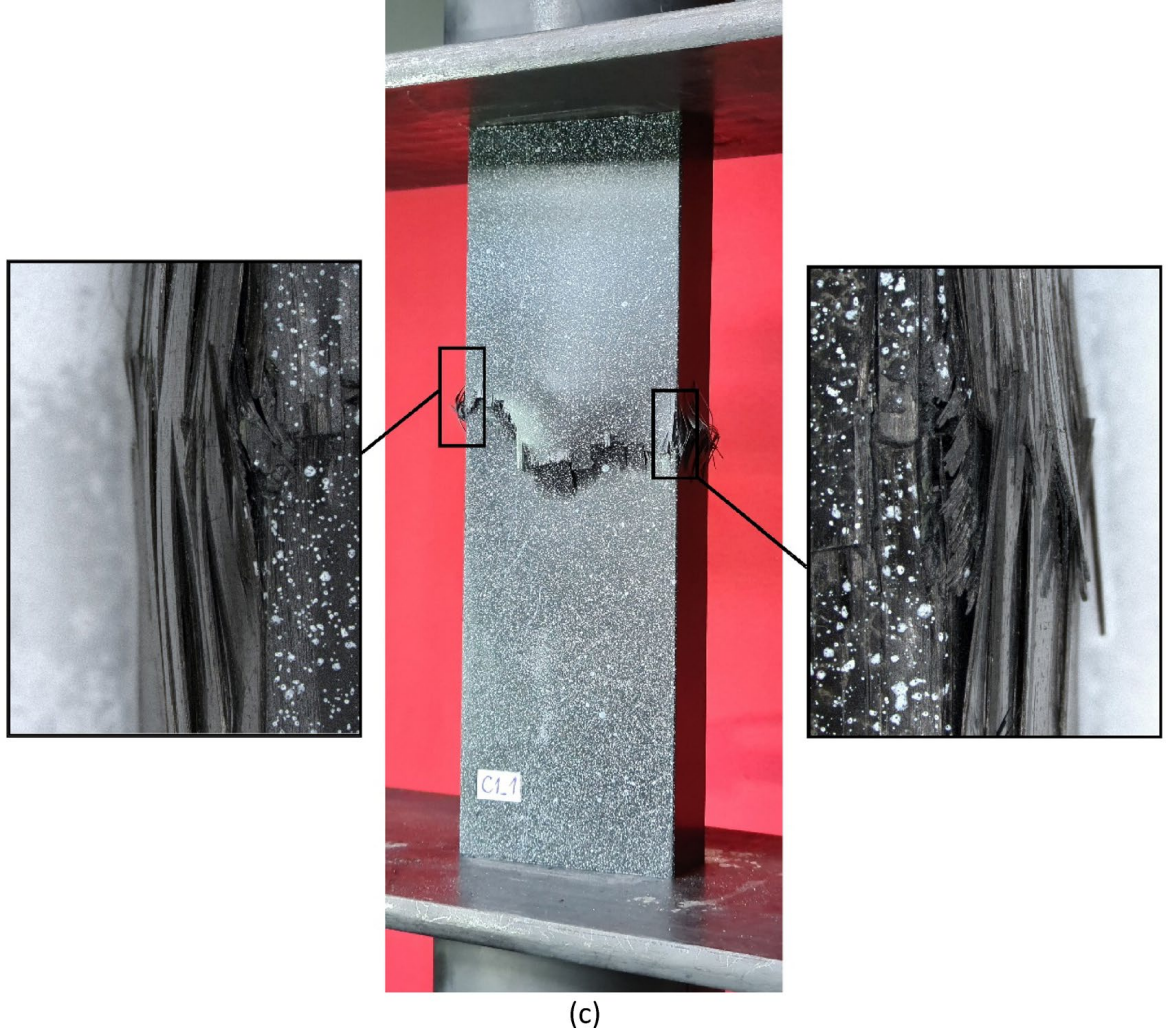
Graphical representation of the failure phenomenon using digital microscopy for highlighting the forms of failure: (**a**) A1-type specimen, (**b**) B1-type specimen, (**c**) C1-type specimen.

In addition, it was decided to conduct a more detailed observation of the failure phenomenon using a digital microscope (Figs. 15 and 16). Accordingly, observations of the failure area were made at varying tilt angles of the microscope head (Fig. 15).

The failure forms obtained using digital microscopy (20x zoom) are presented for three profiles with different cross-section shapes and identical composite lay-up (A1_1, B1_1, C1_1) – Fig. 16. In the study, the microscope head was tilted 10 degrees from the vertical base position. The observation angle set in this way allowed a more in-depth analysis of the damage mechanism of the composite material structure. In addition, an option for depth of field was set to improve the clarity of observation of the damaged area of the composite. This consisted of taking a series of images at a high zoom level of the head relative to the observed test specimens, after which the head was zoomed out while continuing to record images. The result was a single image that maintained sharpness within the damage field under study. A large depth of field is particularly useful, if not crucial, when observations are made at an angle.

Microscopic observations indicated that the failure mechanism was similar across the studied profiles. In the cases studied, both delamination between the constituent plies of the composite and their fracture (dependent on the composite lay-up) were observed. During the study, it was noted that delamination usually initiated failure, and, upon further loading of the structure, fracture of the plies in the delamination area also occurred. Numerical simulations confirmed that delamination initiated the complex failure mechanism, with the Hashin criterion indicating that, in most cases, matrix tension-initiated failure (parameter HSNMTCRT). Due to the occurrence of delamination and ply fracture, further research activities, in the context of numerical simulations, will focus on the development of complex numerical models that incorporate the cohesive zone model (CZM) for delamination modelling and the XFEM technique for ply fracture. In future studies, a high-speed camera will be used to capture the loss of the load-carrying phase.

The dominant failure mode at the time of loss of load-carrying capacity was usually matrix tension and in-plane shear, as verified in the FEM simulation stage. During experimental studies, it was noted that, in many cases, there was no loss of load-carrying capacity; in other cases, delamination occurred, after which the plies in the delamination area were fractured. This paper provides a summary of the research activities carried out as part of the National Science Centre project No. 2021/41/B/ST8/00148 concerning the stability and failure of compressed thin-walled composite profiles with closed cross-sections. Previous papers provided rather limited information on selected profiles, with less analysis of their load-carrying capacity. Previously, most of the research available in the literature concerned composite structures with open cross-sections. The novelty of this paper is a comprehensive summary of the load-carrying capacity results of all thin-walled composite profiles with closed cross-sections tested as part of the research project, both in terms of the influence of the laminate layer arrangement and the cross-sectional shape, based on several interdisciplinary research methods. This paper presents a summary of the behaviour of thin-walled composite columns at the loss of load-carrying capacity, with the failure phenomenon described in quantitative (limit loads) and qualitative (failure modes with precise location) terms for composite structures with various closed cross-sectional shapes.

## Conclusions

Based on the conducted experimental-numerical studies, the load capacity of thin-walled composite structures with closed sections was determined. The load-carrying capacity of the structures was evaluated primarily based on post-buckling equilibrium paths and the failure forms observed. The study estimated the load-carrying capacity of the structure, with particular focus on the influence of the composite lay-up and the cross-section on load-carrying capacity. Consequently, the following conclusions were made:

- The use of a universal testing machine with an optical deformation measurement system, Aramis, enables the experimental evaluation of the structure’s load capacity.
- Numerical simulations with the use of FEM, together with the consideration of progressive failure analysis, allow a comprehensive evaluation of the failure phenomenon with the determination of the post-buckling equilibrium paths and the determination of the damage mechanism (in each ply separately).
- The cross-sectional shape for a particular composite lay-up has a minor effect on the failure loads.
- The composite lay-up for a particular cross-section of the structure has a significant effect on the failure loads.
- The most extensive failure occurred in the extreme (outermost) plies of the composite material.
- In all cases analysed, a complex failure mechanism occurred, including both composite material ply fracture and delamination.

The above conclusions provide a basis for further research into the in-depth analysis of the limit states of behaviour of thin-walled composite structures with closed sections under axial compression. Regarding the complexity of the failure phenomenon, further work will focus, among other things, on the development of more advanced failure models that take into account, in particular, the possibility of simulating the fracture of composite plies and delamination^38^. The present paper was mainly concerned with comparing the failure phenomenon between various cross-section shapes and composite lay-ups in terms of load-carrying capacity, both in quantitative and qualitative evaluations. In the future, it is also planned to compare the obtained results with profiles from isotropic materials and those made using 3D printing technology. Composite materials demonstrate a special characteristic of working mainly in the elastic range, almost to the phenomenon of failure, which will distinguish them from the behaviour of most other types of materials. Further studies will consider activities aimed at comparing the behaviour of the analysed thin-walled composite structures with similar structures manufactured from other types of materials.

## Data Availability

Data Availability Statement: The data that support the findings of this study are available from the corresponding author, upon reasonable request.
